# New Strategies for Stem Cell Mobilization

**DOI:** 10.4084/MJHID.2012.066

**Published:** 2012-10-03

**Authors:** Roberto M. Lemoli

**Affiliations:** Department of Hematology and Oncological Sciences “L. e A. Seràgnoli”, Institute of Hematology, University of Bologna.

## Abstract

Mobilized peripheral blood (PB) is widely used as source of stem cells (PBSCs) for autologous stem cell transplantation (ASCT). The use of cytokines, alone or in combination with chemotherapy (chemomobilization), is the most common strategy applied to mobilize and collect PBSCs. However, a significant proportion of cancer patients fail to mobilize enough PBSCs to proceed to ASCT. Plerixafor is a small molecule that reversibly and transiently disrupts the interaction between the chemokine receptor CXCR4 and its ligand CXCL12 (formerly known as stroma derived factor 1, SDF-1) leading to the rapid release of CD34^+^ hematopoietic stem cells from the bone marrow (BM) to PB. Plerixafor has been recently approved to enhance PBSC mobilization in adult patients with multiple myeloma or non-Hodgkin lymphoma and has been shown to be more effective than G-CSF alone. There is limited experience on combining plerixafor with chemotherapy plus G-CSF in patients who mobilize poorly. Current evidence suggests that the addition of plerixafor is safe and effective in the large majority of the patients with low blood CD34^+^ cell count after mobilization and/or poor yield after the first collection(s). Circulating CD34^+^ cells can be increased by several folds with plerixafor and the majority of the patients considered “poor mobilizers” can be successfully collected. Overall, its mechanism of action inducing the rapid release of CD34^+^ cells from the BM to the circulation makes plerixafor suitable for the ‘preemptive’ use in patients who are hard-to-mobilize.

## Clinical Background

Autologous stem cell transplantation (ASCT) is widely used for the treatment of hematological malignancies. The most common indications are multiple myeloma (MM) and non-Hodgkin lymphoma (NHL) followed by Hodgkin’s disease (HD).^1^ The vast majority of ASCTs are performed with the support of peripheral blood stem cells (PBSCs), thus making their mobilization and collection an important part of ASCT. In fact, the rapid and sustained recovery of the hematopoietic function after ASCT correlates with the number of CD34^+^ hematopoietic stem cells infused.^2^

CD34^+^ cells reside mainly in the bone marrow (BM) niche(s) but they can be effectively mobilized to peripheral blood (PB) by the administration of growth factors such as granulocyte colony-stimulating factor (G-CSF) (filgrastim, lenograstim, pegfilgrastim) or granulocyte-macrophage colony-stimulating factor (GM-CSF) (sargramostim) alone or combined with disease-specific chemotherapy (chemomobilization).^3^,^4^ The minimum dose of CD34^+^ cells to provide a high likelihood of successful engraftment is generally considered to be ≥ 2 × 10^6^ cells/kg^5^,^6^ whereas the ‘optimal’ number of PBSCs for transplantation is 4–6 × 10^6^ CD34^+^ cells/kg in both adult and pediatric patients.^7^ The finding that higher numbers of re-infused CD34^+^ cells have been correlated, at least in some studies,^8^,^9^ with earlier engraftment after transplantation and with better disease-free and overall survival than lower cell doses, has led many transplant centres to attempt the collection of the optimal PBSC number (‘target cell dose’) rather than the minimum dose.

CD34^+^ stem/progenitor cell collection correlates with the absolute number of circulating CD34^+^ cells prior to the apheresis. Peak mobilization after G-CSF alone usually occurs 4–5 days after the initiation of G-CSF,^10^ whereas peak mobilization following chemotherapy-based regimens is more variable and may occur 10–20 days from the start of chemotherapy.

A significant proportion of cancer patients eligible for ASCT fails to mobilize a sufficient number of CD34^+^ hematopoietic stem/progenitor cells due to various pre-mobilization (predictive) factors such as prior treatment with stem cell toxic drugs, underlying disease, age, prior radiotherapy and BM involvement. The failure rate with current strategies in adults is estimated to range from 5% to 40%^3^,^11^,^12^ leading to repeated apheresis sessions, suboptimal grafts associated with delayed hematopoietic recovery, need for re-mobilization and, sometimes, to treatments other than ASCT. The percentage of “poor mobilizers” across different studies is variable depending on definitions, disease categories and lack of standard mobilization and collection practices, so that there are no commonly accepted criteria to define the success/failure rates. Thus, there is a medical need of more effective mobilization strategies for patients with advanced or relapsed lymphomas or patients with MM who may be successfully treated with high-dose chemotherapy followed by ASCT.

## Strategies for PBSC mobilization: risks and benefits

### Growth Factors

G-CSF (e.g., filgrastim, lenograstim) are the only approved mobilization agents in Europe for both adult and pediatric patients. Recent data demonstrate that over 80% to 90% of all ASCT worldwide are performed using either cytokine- or chemotherapy - followed - by - cytokine - mobilized PBSCs.^13^

### G-CSF Alone

The approved dosing for non-pegylated G-CSF for stem cell mobilization is 10 μg/kg s.c., although some investigators use it at higher doses (i.e., up to 32 μg/kg s.c. daily) to rescue poor mobilizers. G-CSF is initiated 4 days prior to the first apheresis session and its administration is continued until the last day of apheresis. CD34^+^ cell levels in the blood usually peak on the fifth day of G-CSF.^10^ The reported total yield of collected CD34^+^ cells across a number of controlled studies ranged from 2.5 to 5.8 × 10^6^/kg (median values) during a median of two to five apheresis sessions. The addition of chemotherapy to G-CSF increases yields at the expense of more side-effects, although the reported failure rates (defined as CD34^+^ cell yields of <2.0 × 10^6^/kg) are not different between the two treatments, with failures rates of up to 23%. After transplantation, the median time to granulocyte engraftment with G-CSF alone has been reported to be 11 days, and for platelet engraftment approximately 11–14 days.^3^

G-CSF is generally well tolerated. Common side effects include bone pain, headache, anaemia and decreased platelet counts. Rare but potentially fatal splenic rupture has also been reported.^14^ In addition, screening for thrombophilia is recommended in normal donors who report a familiar or personal history of previous thrombosis due to some suggestions of thrombotic events during PBSC mobilization with G-CSF in healthy donors.^15^

### Pegylated Granulocyte Colony-Stimulating Factor (Pegylated G-CSF)

The potential of the pegylated form of G-CSF (pegfilgrastim), a longer-lasting variant of G-CSF, to mobilize PBSCs has been investigated in clinical trials.^16^ Its long plasma half-life of 33 hours makes a single dose sufficient to induce stem cell mobilization, whereas G-CSF with a plasma half-life of 3 to 4 hours must be administered daily. The safety profile of pegfilgrastim is similar to that of G-CSF and like all current mobilization methods there is a significant failure rate of around 25%.^17^ Interestingly, both filgrastim and pegfilgrastim are widely recognized as regulators of the immune system by mainly inducing modulatory cells.^18^

### Granulocyte-Macrophage Colony-Stimulating Factor (GM-CSF)

GM-CSF is used less often than G-CSF, and only in USA, for PBSC mobilization because it is less efficient (both when given alone and in combination with chemotherapy) and has a more unfavourable safety and tolerability profile than G-CSF.^3^,^13^ GM-CSF is sometimes used in combination with G-CSF in patients who failed an initial mobilization attempt.

### Chemomobilization

Most mobilization regimens combine treatment with G-CSF (and rarely GM-CSF) after administration of a disease-specific chemotherapy regimen to achieve higher CD34^+^ cell yields than treatment with G-CSF alone, both in patients with MM and NHL (although failure rates with G-CSF plus chemotherapy seem to be as high as with G-CSF alone).^3^,^11^ For instance, Moskowitz et al.^19^ reported that mobilization with G-CSF alone (10 μg/kg daily) yielded 1.5 × 10^6^/Kg CD34^+^ cells compared with 6.7 × 10^6^/Kg CD34^+^ cells when chemotherapy plus G-CSF were used. Additional benefits of chemomobilization include fewer number of required apheresis sessions compared to G-CSF alone. More importantly, there is indication that chemomobilization, particularly in lymphoma, reduces, in vivo, the tumour load and tumour cell contamination in the apheresis product. In fact, PBSC mobilization is often part of a cycle of induction or salvage treatment for lymphoma patients thus avoiding additional costs and risks associated with the use of unnecessary chemotherapy for mobilization. Chemomobilization is also commonly used in MM using a single dose of cyclophosphamide. In this case, the benefit of higher cell yields (than with G-CSF alone) may be offset by less predictability of timing and an increased risk for the patient (i.e., increased morbidity, greater risk of infection and febrile neutropenia, more hospital admissions, transfusions, antibiotic therapy, and drug-specific toxicities) without any well documented anti-tumor effect.^20^

One potential problem related to the use of chemotherapy is that PBSC mobilization is less predictable and may vary substantially between patients. Thus, is it necessary to monitor leukocytes and CD34^+^ cell counts over several days to determine when to begin apheresis.^3^ Overall, the addition of a myelosuppressive regimen to a cytokine may result in a higher cell yield than cytokine alone, but this result needs to be balanced against the increased risks for the patient and the greater resource utilization unless chemotherapy is part of the treatment strategy.

## Definition of “poor mobilizer” and risk factiors

As mentioned, the definition of “poor mobilizer” varies according to different parameters analyzed to evaluate PBSC mobilization: peak of CD34^+^ cells in PB, fold-increase of circulating CD34^+^ cells, CD34^+^ cells collected, number of candidate patients undergoing ASCT. As a consequence, different criteria have been proposed to define a successful PBSC mobilization and the adequate apheresis yield, but these data are difficult to analyze and compare to each other.^3^,^11^,^12^,^21^ The extensive review of predictive factors for poor mobilization is beyond the scope of this article (see 3,6,7,12,19). However, it should be kept in mind that in addition to baseline parameters, during- and post-mobilization factors have been poorly exploited due to the lack, so far, of rescue strategies. For instance, febrile neutropenia is one major complication after administration of mobilizing chemotherapy.^20^ The release of pro-inflammatory cytokines may negatively affect stem cell proliferation and mobilization. Furthermore, genetic factors as well as polymorphisms in cytokine gene receptors are believed to be responsible for the great variability in mobilization responses in allogeneic donors.^22^

Need for supportive care such as antibiotics for febrile neutropenia and blood product support is associated with mobilization failure in patients with NHL.^23^ Moreover, in patients receiving chemomobilization, slow leukocyte and platelet recovery as well as anemia indicate poor marrow function. However, type and dose of chemotherapy may influence the risk of mobilization failure as severe thrombocytopenia induced by alkylating agents administered during mobilization can be a risk factor for mobilization failure while high-dose cytarabine mobilization regimen often induces severe thrombocytopenia and neutropenia without negatively affecting stem cell mobilization.^24^

Other factors predicting mobilization failure are: delayed or anticipated timing of apheresis (due to insufficient circulating stem cells monitoring) and/or small volume of processed blood which may affect PBSC collection even in patients showing a satisfactory peak of CD34^+^ cells in the PB.

For these reasons, a working group promoted by GITMO (Italian Group for Stem Cell Transplantation) proposed the definition of “poor mobilizer” identifying “proven poor mobilizer” and “predicted poor mobilizer”.^25^ In order to develop criteria for the definition of “poor mobilizer”, the working group used the analytic hierarchy process (AHP) which had been developed to establish priorities and to make the best decision when both the quantitative and qualitative aspects of a decision need to be considered and a poor information base is available. AHP is a multistep process that includes four major phases: 1) defining the goal; 2) decomposing the problem and identifying critical issues; 3) categorizing/framing the main criteria; 4) defining a hierarchy of the criteria.

GITMO panel selected two conceptual criteria to identify the “proven poor mobilizer”: the peak of circulating CD34^+^ cells during mobilization and the absolute number of harvested CD34^+^ cells. All participants agreed that pre-apheresis CD34^+^ count in PB is the best predictor of CD34^+^ cells in the aphaeresis products^11^,^26^–^30^ and, operationally, considered a peak of CD34^+^ cells >20 μl in PB, as a reliable indicator of a satisfactory mobilization ability. Moreover, the GITMO panel identified 2.0×10^6^ CD34^+^ cells/kg as the minimum safe dose for ensuring rapid neutrophil and platelet recovery both in lymphoma and in MM patients to be achieved with a maximum number of 3 aphereses.^11^,^12^,^31^ These parameters and indicators applied to both chemomobilization and G-CSF alone strategies although the timing of CD34^+^ cells peak and doses of G-CSF are different and should be considered.^25^

Furthermore, GITMO panel selected 3 major and 5 minor criteria to identify the “predicted poor mobilizer”.^25^ The most important criteria were felt to be: previous cytotoxic chemotherapy, irradiation on BM bearing bones and failure of previous mobilization attempt.

Among the other factors associated with unsuccessful mobilization, GITMO panel selected advanced phase disease (i.e. at least 2 prior cytotoxic lines), refractory disease, extensive BM involvement at mobilization, BM cellularity <30% at mobilization and age >65 years as minor criteria. The proposed definitions should be validated in prospective clinical studies.

In conclusion, poor mobilization of PBSCs is a major limitation for patients eligible for ASCT. The availability of new drugs, aimed at optimizing PBSC mobilization, requires a stringent definition of “poor mobilization”. In this view, GITMO panel recommended that patients previously failing at least one mobilization attempt should be candidate for new mobilizing strategies. In addition, the use of standard criteria for identifying both the “proven and the predicted poor mobilizer” before planning the use of new mobilizing agents was recommended. To this end, the GITMO working group tried to define simple, but stringent operational criteria for the identification/prediction of “poor mobilizer” in the setting of lymphoproliferative diseases.

## New approaches to optimize HSC mobilization

In adult life, the chemokine receptor CXCR4 and its ligand stromal cell-derived factor-1 (SDF-1/CXCL12) are critically regulating the retention of hematopoietic stem cells in the BM. Under physiological conditions (i.e. in absence of “danger signals”) the release of hematopoietic stem cells from the BM occurs infrequently and follows a circadian loop. Tissue damage, infections or flogosis induce the exit of stem cells from the BM to contribute to tissue repair.^32^ Disruption of the CXCR4/CXCL12 axis in the BM, which can be directly achieved by CXCR4 antagonists or indirectly by G-CSF through the development of a proteolytic enviroment, increases the motility of hematopoietic stem cells and their egress from the BM.^33^

Plerixafor (formerly AMD3100) is a CXCR4 chemokine antagonist that has been shown to increase the number of circulating CD34^+^ cells in healthy volunteers and cancer patients alone or with G-CSF.^34^,^35^ The key feature of this chemokine receptor/ligand interaction is the rapidity of the mobilization process and stands in clear contrast with G-CSF-based mobilization where up to four days of treatment are required before the significant increase of circulating CD34^+^ cells is observed.^36^ Consistent with its antagonistic activity on the CXCR4 receptor, plerixafor also increases the number of circulating leukocytes. There are two distinct phases of stem cell mobilization according to different routes of administration: the peak occurs approximately four hours after intravenous injection while 10–12 hours are required for stem cell release from the BM after subcutaneous administration. By 24 hours the mobilizing effect of plerixafor is returned to baseline or close to baseline.^37^ Therefore, the rapid biological activity of plerixafor allows its administration “on demand” without planning the timing of administration in advance.

Early studies in patients with NHL and MM suggested the superiority of G-CSF plus plerixafor over G-CSF alone in regard to mobilization efficiency.^38^ More recently, a compassionate use study including 115 patients who had failed at least one previous mobilization attempt showed a success rate for re-mobilization with G-CSF plus plerixafor of 60% for NHL, 71% for MM and 76% for HD.^39^ Similar results have been shown in an European compassionate use study including 56 patients with lymphoma or MM, where the success rate was 75%.^40^

In two phase III randomized placebo-controlled studies in MM^41^ and NHL patients,^42^ the combination of G-CSF plus plerixafor was found to be safe and superior in terms of mobilization efficacy as compared to G-CSF plus placebo. In MM patients randomized to G-CSF plus plerixafor, 71.6% of the patients achieved the primary study endpoint (collection of at least 6 × 10^6^/kg CD34^+^ cells with less or equal to two aphereses) compared to only 34.4% of patients receiving G-CSF and placebo.^41^ Similarly, 59% of NHL patients achieved the primary study endpoint (collection of at least 5 × 10^6^/kg CD34^+^ cells with less or equal to four aphereses) compared to only 19.6% of patients mobilized with G-CSF plus placebo.^42^ Plerixafor-mobilized PBSCs did show rapid and sustained engraftment after high-dose therapy in both studies. Noteworthy, Maziarz and co-workers^43^ performed a post-hoc analysis based on data from the randomized trial of plerixafor + G-CSF vs. placebo + G-CSF in NHL patients. The investigators evaluated the efficacy of the addition of plerixafor to G-CSF on the evening of day 4 in patients with pre-plerixafor circulating CD34^+^ cell count < 10 × 10^6^/l, to achieve the collection of the minimum (≥ 2 × 10^6^/kg) or the target (≥ 5 × 10^6^/kg) cell dose. The results demonstrated that patients who had been randomized to receive plerixafor in addition to G-CSF showed a 6 fold-increase of PB CD34^+^ cells on day 5 compared to only 1.6 fold-increase in patients receiving G-CSF and placebo. These data resulted in a significantly higher cumulative number of CD34^+^ cells after 2 apheresis days in plerixafor-treated patients as compared to placebo patients (2.92 vs. 0.94 × 10^6^/kg). Overall, 78% of patients in the plerixafor + G-CSF group achieved the primary end point compared to only 34.2% in the control group. Thus, the addition of plerixafor to G-CSF enabled the collection of the minimal transplantable dose in the majority of patients with a PB CD34^+^ cell count < 10 × 10^6^/L on day 4. A statistically significant increase in PBSC collections was also obtained in patients mobilized with G-CSF plus plerixafor and with PB CD34^+^ count < 20 × 10^6^/L on day 4.^43^ Taken together, these results provide a clear example of the potential of ‘early intervention’ with novel strategies to rescue cancer patients who can be considered ‘proven poor mobilizers’ as they have < 10–20 × 10^6^/L PB CD34^+^ cells at the peak time of mobilization after G-CSF mobilization. However, clinical studies involving the use of Plerixafor in children are needed to confirm its potential for PBSC mobilization in this patient population.

## Plerixaflor combined with chemomobilization

At present chemomobilization is considered the mobilization standard in many transplant centres especially in lymphoma patients. However, published results indicate that the addition of chemotherapy to G-CSF does not prevent poor mobilization.^3^,^11^,^12^ Limited data is available on the effects of the administration of plerixafor added to chemomobilization to enhance the mobilization of PBSCs. Dugan and co-workers^44^ evaluated prospectively the safety and efficacy of plerixafor combined with chemotherapy and G-CSF in an open-label, multicenter trial. In this study, 40 patients (26 MM, 14 NHL) received various chemotherapy regimens followed by G-CSF plus plerixafor. The mean fold-increase of PB CD34^+^ cells was 1.7 fold after plerixafor. The combination was well-tolerated. However, based on the results published on the peak number of circulating CD34^+^ cells and apheresis yields, most of the patients could not be considered as hard-to-mobilize as pre-plerixafor median PB CD34^+^ counts were 33 × 10^6^/L in NHL patients and 150 × 10^6^/L in MM patients, respectively. Recently, the addition of plerixafor to chemotherapy plus G-CSF mobilization was tested in patients who mobilize poorly (i.e. re-mobilization or first mobilization with low blood CD34^+^ counts or poor collection yields).^45^–^48^ Based on the mechanism of action, plerixafor causes a rapid release (5–11 hours) of CD34^+^ cells from the BM to circulation, which makes the drug suitable for pre-emptive or ‘on demand’ use in patients who are hard-to-mobilize. Patients were classified as ‘poor mobilizers’ based on daily monitoring of PB CD34^+^ cell counts during the recovery phase after chemotherapy and G-CSF and/or the collection of PBSCs was felt to be inadequate to proceed to ASCT. By considering only the studies with more detailed information available,^40^–^43^ 28 out of 34 patients (85%) collected ≥ 2 × 10^6^/kg CD34^+^ cells after the first mobilization attempt with a median of two plerixafor injections.

The analysis of published data suggests that in poor mobilizers plerixafor may not be effective in inducing CD34^+^ cell mobilization when the leukocyte count is very low. Therefore, the critical issue of the optimal timing for plerixafor addition cannot be addressed conclusively. Too early addition of plerixafor may not be cost-effective as many patients may be successfully collected by waiting 1–2 days especially if PB CD34^+^ cell and leukocyte counts are rising. On the other hand, waiting too long may be deleterious as the mobilization induced by chemotherapy plus G-CSF may diminish and hence late addition of plerixafor might be less effective. Thus, future studies should test prospectively well-defined algorithms, perhaps based on leukocyte and CD34^+^ cell counts and/or the results of first day collection, to optimize the use of plerixafor after chemomobilization.

Altogether, few patients reported in four small series^45^–^48^ mobilized with chemotherapy/G-CSF plus plerixafor have been transplanted so far. Only two patients, who received grafts containing 1.8 and 2.1 × 10^6^ CD34^+^ cells/kg, respectively, were reported to have slow platelet engraftment. In the German series,^48^ all 24 patients mobilized with chemomobilization plus plerixafor engrafted. Thus, based on the study by Dugan et al.^44^ and five patient series on add-on use of plerixafor after chemomobilization, this combination appears to be safe and no major side-effects attributable to plerixafor have been reported. In addition, plerixafor-mobilized PBSCs did show rapid and sustained engraftment. This finding supports the data from randomized phase III studies showing that patients mobilized with G-CSF plus plerixafor have stable and sustained engraftment after high-dose therapy.

Beside stem cell mobilization, few additional topics related to plerixafor administration should be mentioned. It is known that mobilization with G-CSF plus plerixafor results in different graft composition when compared to G-CSF alone mobilization, including more CD34^+^CD38^−^ cells^49^ as well as more NK-cells and T cells.^50^ There are no data on the graft content, other than CD34^+^ cell dose in patients mobilized with a combination of chemotherapy, G-CSF and plerixafor. As graft content may be of importance also for immune reconstitution and long-term patient outcomes, this issue deserves further studies. Furthermore, available data indicates that the use of plerixafor is not associated with increased mobilization of tumour cells in myeloma or lymphoma patients.^51^,^52^

Plerixafor is not recommended in patients with acute myeloid leukemia due to the mobilization of leukemic cells into circulation. The activity of plerixafor on BM microenviroment and the subsequent release of leukemic cells has been recently exploited therapeutically to enhance the antileukemic effect of conventional chemotherapy in resistant/relapsed patients (chemosensitization).^53^

## Conclusion

Plerixafor is an effective and well-tolerated novel drug for PBSC mobilization in adult patients with lymphomas and MM who are planned for high-dose chemotherapy. While it mobilizes CD34^+^ cells on its own, plerixafor significantly improves the mobilization capacity of G-CSF when the two drugs are used in combination.

In addition, although the experience of adding plerixafor to chemomobilization in hard-to-mobilize patients is limited, early results have shown that this combination is feasible and safe and is able to improve stem cell mobilization by several fold to facilitate timely collection and subsequent ASCT. The vast majority of the patients reported so far^54^ achieved the minimum collection target and could proceed to ASCT. Of note, according to the proposed definition of “poor mobilizers”^25^ most of these patients may be included in this patient group. At present, there is no available prospective data on chemomobilization and plerixafor in “predicted poor mobilizers”.

However, one of the main issues in this setting remains patient selection and timing of plerixafor administration. Available evidence based on small case numbers^54^ suggests that the beneficial effect of plerixafor after chemomobilization may not be seen with very low blood leukocyte counts although no clear algorithms can be presented due to scanty information available. Conversely, based on available data, plerixafor may be added to patients with low PB CD34^+^ cell counts (e.g. < 10 × 10^6^/L) at the time of hematopoietic recovery after chemotherapy (e.g. blood leukocytes > 5 ×10^9^/L) or to patients with poor first collection yield (e.g. < 1 × 10^6^ CD34^+^ cells/Kg) (“proven poor mobilizers”). On this basis, the preemptive use of plerixafor may be the most cost-effective way to use this new drug by preventing mobilization failure and need for re-mobilization or marrow harvest and, perhaps, resulting in a higher number of lymphoma and MM patients submitted to ASCT.^54^

Overall, the decision to add plerixafor to chemotherapy should be based on clinical parameters taking into account predictive criteria (e.g., age, disease status, prior lines of chemotherapy and prior radiation therapy) as well as “actual” criteria such as the absolute PB CD34^+^ cell and leukocyte counts 1 day prior or on the day of PBSC collection and the overall therapeutic goal (e.g., sufficient CD34^+^ cells for single or double transplantation). In this view, the patient-adapted use of plerixafor in adjunct to G-CSF, based on PB CD34^+^ count and target cell dose, has been recently proposed by Costa and co-workers^55^ and resulted in a safer and more efficient collection of PBSCs when compared to cyclophosphamide and G-CSF.^56^

The main potential disadvantage of the pre-emptive use of plerixafor would be the need for real-time CD34^+^ cell count. On the other hand, we may be able to reduce remobilization sessions and the number of apheresis, therefore saving financial resources and avoiding delays in the transplant program. Conversely, the use of plerixafor for stem cell mobilization in “predicted poor mobilizers” is limited by the lack of reliable models to predict mobilization failure. Furthermore, the application of plerixafor to patients who have previously failed stem cell mobilization (“second line” use) involves more aphereses and additional growth factor costs with significant delays for ASCT

Due to common use of chemomobilization in clinical practice, studies evaluating the optimal use of plerixafor in poor mobilizers after chemomobilization should be pursued.
